# Barriers and Solutions to Successful Problem-Based Learning Delivery in Developing Countries – A Literature Review

**DOI:** 10.7759/cureus.43187

**Published:** 2023-08-09

**Authors:** Jhiamluka Solano, Melba Zuniga Gutierrez, Esther Pinel-Guzmán, Génesis Henriquez

**Affiliations:** 1 Cardiology, Scunthorpe General Hospital, North Lincolnshire, GBR; 2 Research and Development, Asociación de Educación Médica Hondureña, Tegucigalpa, HND; 3 Research, Organization for Woman in Science for the Developing World, Tegucigalpa, HND; 4 Medicine, Universidad Católica de Honduras, Tegucigalpa, HND; 5 Medicine, Universidad Nacional Autónoma de Honduras, Tegucigalpa, HND; 6 Medicine, Asociación de Educación Médica Hondureña, Tegucigalpa, HND

**Keywords:** teaching feedback, curriculum development and evaluation, medical education, developing countries, problem-based learning

## Abstract

Problem-based learning (PBL) was introduced in the 1960s as an alternative to traditional teacher-centered and discipline-based preclinical medical education. A literature review was conducted to explore the barriers and solutions to successful PBL uptake and delivery in developing countries. The review involved the search of articles and scientific studies on PubMed, The Lancet, and Scielo. The review focused on the medical education literature, using as a primary search criterion "problem-based learning" in combination with "developing countries" and "education". The search was limited to articles in Spanish and English published between 2011 and November 2021, except for three articles due to their relevance to the subject. Faculty development programs are the cornerstone when implementing a new methodology in developing countries. Early career development, PBL methodology, and the available assessment options should be the primary learning objectives of these programs. Stakeholders will need to plan using available resources following the experience of other countries and institutions encouraging collaborative development. Evaluation and assessment will be crucial to understand the impact of PBL, and considerations should be taken to implement an integrated curriculum. Medical Education Research should be encouraged, appraised, and disseminated to improve evidence-based decision-making, creating a constant development cycle. PBL is innovative and represents many unanswered questions that will develop in the following decade as more schools implement new methodologies and Research on PBL.

## Introduction and background

Problem-Based Learning (PBL), introduced to preclinical medical education in 1969, was defined as "an instructional (and curricular) learner-centered approach that empowered learners to conduct research, integrate theory and practice, and apply knowledge and skills to develop a viable solution to a defined problem" [1,2]. Extensive research has developed since the introduction of PBL, creating an abundant source of discussion in medical education [1].

Strong scientific evidence supports PBL as an exceptional tool for developing an efficient approach to clinical challenges. It encourages critical thinking skills rather than factual knowledge through Self-Directed Learning (SDL), reflective practice, and formative feedback [3,4]. Medical schools in developing countries have struggled to adopt new methodologies in their curriculum. The available literature regarding its implementation in settings with limited resources appears sparse [5].

The following literature review outlines the main challenges and potential solutions for successful PBL delivery to guide developing countries to transition successfully from the traditional teacher-centered approach exploring the basic concepts and the advantages and disadvantages of PBL.

## Review

Methodology

The review focused on medical education literature primarily in PubMed; the search terms used were Medical Education, Problem-based Learning, and developing countries. We used AND and OR as our Boolean operators. We included literature reviews, Meta-analyses, Randomized Controlled Trials, and systematic reviews published in English between 2011 and 2021. However, articles published before 2011 were considered if they offered relevant data to improve the development of the research question. We excluded articles that were not original studies, book chapters, published in other languages, unrelated to medical education, and outside the year interval with the abovementioned exception.

**Figure 1:**
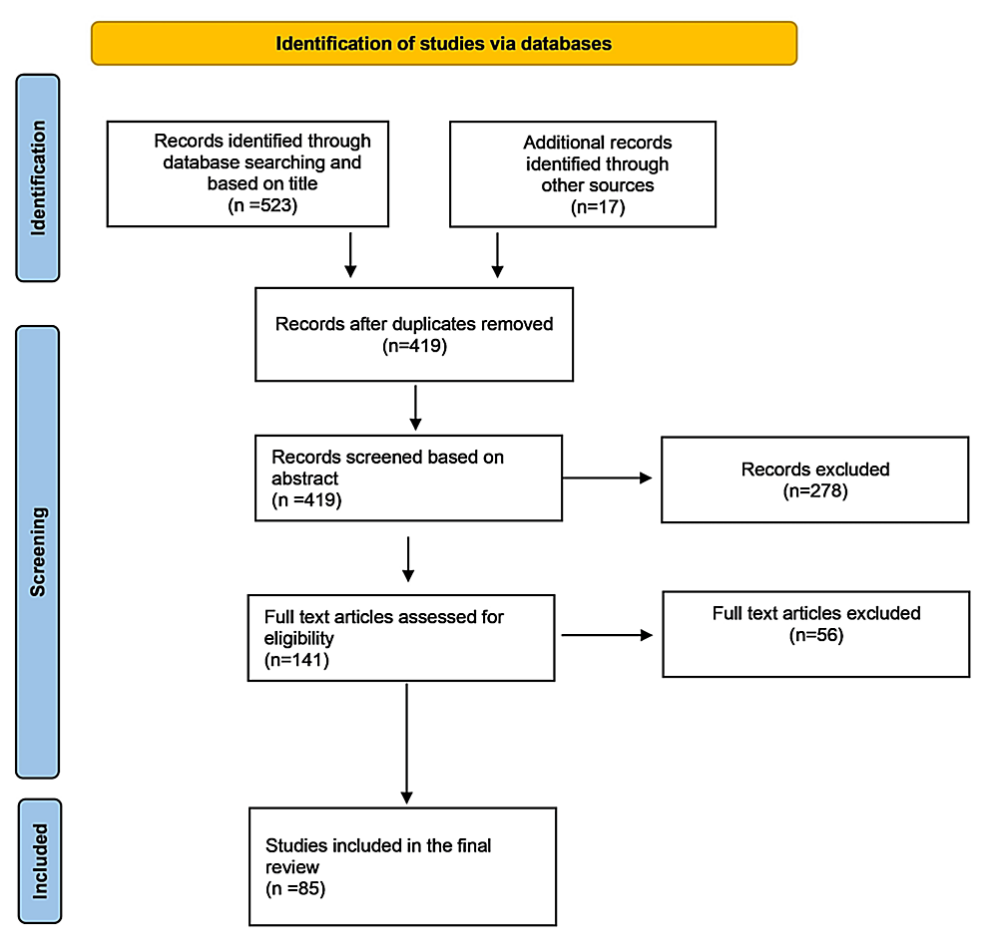
PRISMA flowchart.

During the first stage of the selection process, we found 873 articles related to the search terms used. We selected 523 based on the title, and a further 17 were found through other search engines. We identified 121 duplicate articles and appraised the remaining articles based on the abstract. We excluded 278 articles before the last stage of the selection process; the remaining 141 articles' full text was appraised to select a final sample of 85 articles. A PRISMA diagram shows the search, identification, screening, and review process (Figure 1).

Problem-Based Learning

PBL is an instructional method that involves presenting the problem, solving it using clinical reasoning skills, re-addressing the problem with gained knowledge, and concluding by summarising what has been learned [6,3]. PBL has evidence supporting its effectiveness compared to traditional teaching methods [7-11]. Assessments in PBL can vary depending on the learning objectives, including portfolios, objective structured clinical examinations (OSCE), written assessments, extended matching questions, and Progressive Disclosure Questions [3].

Advantages and Disadvantages of PBL

The benefits for learners include the development of critical thinking, knowledge acquisition, and teamwork skills; students also achieve better exam scores. As for tutors, the advantages encompass the standardized and communicative environment. The disadvantages include the narrow subjects it can be applied to in the curriculum, the need for more students than qualified teachers, and the challenging transition from traditional methods [8-23].

Medical Education Challenges in Developing Countries

Developing countries face challenges such as corruption, underfunding and bureaucratic barriers that harm healthcare and medical training [24-36]. Unfortunately, this leads to a brain drain where the best professionals leave and never return, leaving medical education to fail and perpetuating faults [37]. During the pandemic, this was more evident as a pitfall due to the challenges of adapting to the new normal [38-43]. This leaves a significant gap in the development of future doctors as a poor understanding of medical education makes it challenging to implement new methodologies leaving a teacher-centered traditional curriculum [2,44,45].

Discussion

The need for an innovative curriculum has been continuously highlighted in many studies [15,46-48,9]. There are some recommended elements to accomplish the implementation in limited resources settings:

Faculty Development Programmes

The evidence suggests the role and importance of faculty development programs [49,4,50,42]. A sustainable program should aim to train their local Faculty and use funding only for external experts when needed. Peer mentoring programs can improve faculty training. The first generation becomes future generations' mentors, creating a long-term sustainable program. Furthermore, we should encourage medical schools to have medical education departments [44] that explore new methodologies and their impact on student learning [51].

Early Educators Career Development

The lack of medical education departments promotes isolated efforts for competent facilitators' development. The experience in medical education can be limited before becoming an educator [52]. Early career development in medical education will be essential to allow future educators to develop the required skills. Medical students and postgraduate trainees will represent the primary resource of mentees [53]. Overcoming the cultural barrier the medical hierarchy represents is vital. We can follow a faculty milestone form to guide faculty members' development by assessing six core competencies: patient care, medical knowledge, system-based practice, practice-based learning, improved professionalism, and communication [54]. Junior educators will represent the new generation to break the cycle and promote development.

Overcoming Resource Limitations

Medical Education needs to evolve to meet the needs and requirements of students [55]. However, there is not much literature regarding the effective use of available resources in medical education to improve its quality. Most sectors have adequate resources to develop sustainable Programmes [56]. Limited funding accounts for understaffed departments, equipped facilities, lack of training courses, external evaluation, and scientific information [57]. However, there are mechanisms to achieve positive outcomes without funding through grants or free access from developed countries [58].

Moreover, there are different types of rationing in Medical Education [59]. Using a single tutor can be helpful when Faculty Development Programmes are created, and no local experts are available. Not an ideal solution, but it can help. The use of flipped classrooms can be helpful for students in understanding and learning at their own pace and revisiting after [51]. Furthermore, we can use an approach based on four economic principles: targeted talent approach, use of the internet as a network tool to broaden the scope, dissemination of knowledge of PBL, and extrapolation of the successful approaches efficiently [57].

Inter-Institutional Cooperation

Developed countries often collaborate with medical schools in underdeveloped countries. This is an opportunity to access resources and develop based on the experience of others [60]. Different institutions' frameworks can aid stakeholders in identifying objectives, deliverables, funding flow, management principles, promoting standards, developing medical educators constantly, and disbursement procedures that can be improved [61,62]. Finally, governments and universities are essential in designing training plans, ultimately impacting healthcare delivery in different stages [26].

Assessment

Traditional teaching methods have been distorted from the original principles by insufficient training for trainers in underdeveloped countries. Introducing PBL becomes a teaching and assessment challenge. Although most literature aims for formative assessment used in PBL, summative assessment can also be used when adequately designed. On the other hand, peer assessment of professional behaviors can provide a better understanding of the impact of PBL [63]. Peer assessment can be highly reliable for 'within group' comparison but poor for 'across group' comparison [64]. The self-assessment scale can also assess performance and improve SDL and critical thinking by promoting reflective practice [65]. Courses will be crucial to provide educators with different options and enable them to adopt the one they feel most comfortable with.

Evaluation

Evaluation is crucial in every learning process to ensure constant development and feedback. Evaluation requires design and a structured approach to ensure relevant and actionable results. In developing countries, effective evaluation processes are not standard, representing a common cultural and methodological challenge [66]. Pilot faculty development programs can represent a good starting point to understand and develop evaluation knowledge, which can be implemented for evaluation at a larger scale.

Quality in medical education has been categorized into four dimensions: structural and curricular level, procedural aspects of teaching, quality referring to teacher characteristics, and outcomes of teaching activities. Educational structure and processes can be assessed using student feedback questionnaires. There are many ways to evaluate the quality of teaching ("Medical Student Experience Questionnaire"; "Marburger Fragebogen zur Evaluation des Lehrangebots in der Medizin"; "Learning Environment Questionnaire"; and "Medical Instructional Quality"). On the other hand, when considering evaluating individual teachers and their characteristics, we can use the "Stanford Faculty Development Program survey" and the "Student Evaluation of Teaching in Outpatient Clinic". Although inaccurate, postgraduate entry exams can assess the last dimension [67].

Using an Integrated Approach

Liu et al. [11] contrasted Lecture-based Learning (LBL) with integrated curriculums and found that the latter student group had superior knowledge assessment scores. Integrated curriculums show improved comprehension [68,69]. Single-method curriculums should be avoided [15,67,46,47,48]. For this purpose, we encourage the aim of an integrated curriculum [70].

Identifying and understanding local needs through needs assessments is crucial [71,72]. Most studies support PBL over LBL [73,74]. However, Bergman et al. [75] found that PBL affects concept-based courses. LBL helped students retain non-clinical information long-term [76,77]. We should avoid using PBL in subjects like anatomy [78-80]. Gustin et al. [81] found that students' use of deep learning approaches was similar in both curriculums. New generations of medical students are more inclined to use technology when learning; thus, technology is a tool we need to explore further [51].

Medical Education Research

As part of implementing new methodologies, we need to address the impact caused by the learning process. Only research understands the true impact of benefits, challenges, and designs of implementing new strategies [82,83]. Developing countries struggle to produce research, and Medical Education research is a more significant challenge [84]. Once a new methodology is implemented with a following evaluation and research process, the initial outcomes should prompt follow-up studies to encourage development [85]. Most developing countries do not have adequate medical education publications, representing an exceptional opportunity to narrow the knowledge gap in this area [84]. Finally, once research in medical education is established, we must encourage researchers at all experience levels to publish their findings in high-impact journals [86].

## Conclusions

Successful PBL delivery can represent a significant challenge for developing countries. Patient quality of care can improve significantly with better-trained doctors; thus, developing medical education should be an ethical obligation. Implementing PBL will require more than training educators but a cultural and administrative effort from medical schools. Overcoming resource limitations and promoting interinstitutional cooperation are vital steps.

The first and most crucial step in implementing any change to a curriculum is training the educators. Although Medical Education Departments should be the long-term goal, programs can help improve medical education. Additionally, early career development, assessment, and research should be the primary learning objectives of the program to improve evidence-based decision-making. Either members or externals need to design the evaluation process for a later stage in the implementation. Because of the evidence supporting this, we should consider implementing an integrated rather than a PBL-based curriculum.

To our knowledge, this is the first review that addresses all the possible barriers and solutions to successful PBL delivery by exploring the documented experiences in developing countries. After carefully considering the available evidence, we outlined 8 recommended fundamental areas of development to implement PBL. These recommendations will help develop medical education further. The available evidence provides enough knowledge to guide medical schools to successful PBL delivery in developing countries. If medical schools in developing countries do not evolve, they risk becoming obsolete, negatively impacting patient care and professional development outside their frontiers for their students.
